# Crocins for Ischemic Stroke: A Review of Current Evidence

**DOI:** 10.3389/fphar.2022.825842

**Published:** 2022-08-05

**Authors:** Kiran Shahbaz, Dennis Chang, Xian Zhou, Mitchell Low, Sai Wang Seto, Chung Guang Li

**Affiliations:** ^1^ NICM Health Research Institute, Western Sydney University, Penrith, NSW, Australia; ^2^ Reserach Centre for Chinese Medicine Innovation, The Hong Kong Polytechnic University, Kowloon, Hong Kong SAR, China; ^3^ Department of Applied Biology and Chemical Technology, The Hong Kong Polytechnic University, Kowloon, Hong Kong SAR, China

**Keywords:** ischemic stroke, Crocins, saffron, neuroinflammation, antioxidant, molecular targets, toxicity and safety, clinical trial

## Abstract

Crocins (CRs) and the related active constituents derived from *Crocus sativus* L. (Saffron) have demonstrated protective effects against cerebral ischemia and ischemic stroke, with various bioactivities including neuroprotection, anti-neuroinflammation, antioxidant, and cardiovascular protection. Among CRs, crocin (CR) has been shown to act on multiple mechanisms and signaling pathways involved in ischemic stroke, including mitochondrial apoptosis, nuclear factor kappa light chain enhancer of B cells pathway, S100 calcium-binding protein B, interleukin-6 and vascular endothelial growth factor-A. CR is generally safe and well-tolerated. Pharmacokinetic studies indicate that CR has poor bioavailability and needs to convert to crocetin (CC) in order to cross the blood-brain barrier. Clinical studies have shown the efficacy of saffron and CR in treating various conditions, including metabolic syndrome, depression, Alzheimer’s disease, and coronary artery disease. There is evidence supporting CR as a treatment for ischemic stroke, although further studies are needed to confirm their efficacy and safety in clinical settings.

## Graphical Abstract

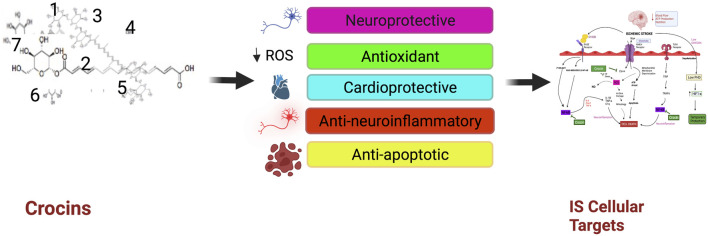

## Introduction

Stroke (cerebral apoplexy) is a serious cerebrovascular disease and the second leading cause of death globally (WHO, 2020). There are three different categories of stroke, namely ischemic stroke (IS), hemorrhagic stroke (Waziry et al., 2020), and transient ischemic attack (Martinez et al., 2020). The prevalence of stroke varies in different countries, with a high incidence in Oceania, Asia, North Africa, and parts of America (Venketasubramanian, 2021; Zhou et al., 2021). Globally, 101.5 million people suffered from a stroke in 2019, causing 6.6 million deaths. Of these, 77.2 million were IS, resulting in 3.3 million deaths (AMA, 2021; Yang et al., 2021).

IS is caused by a blood clot formed in the brain vasculature (thrombotic stroke) or in the peripheral system travelling to the brain through the bloodstream (embolic stroke) (Shiber et al., 2010; Khan et al., 2017; Ye et al., 2021). Currently, there are still limited therapies for IS, either by surgical intervention to remove the thrombus *via* thrombectomy (Yoo and Andersson, 2017) or pharmacological interventions using recombinant tissue plasminogen activator (rtPA) intravenous (IV) and lipid-lowering drugs such as statins (Fuentes et al., 2009; Kazley et al., 2013; Alakbarzade and Pereira, 2020; Barthels and Das, 2020; Wechsler, 2020; Orset et al., 2021). These therapies have limited efficacy (Singh and Singh, 2019), low prognosis, and are associated with adverse reactions and the risk of complications (Zheng et al., 2019; Cui et al., 2020; Pergolizzi Jr et al., 2020). In addition, IS patients often have limited access to rtPA (Barthels and Das, 2020) due to the narrow therapeutic window (which must be administered within 4.5 h of IS onset) (Fukuta et al., 2017; Barthels and Das, 2020). The adverse effects of rtPA, such as hemorrhagic transformation with increased matrix metalloprotein (MMP) (Orset et al., 2021), anaphylaxis and systemic bleeding (Chapman et al., 2014; Khandelwal et al., 2021) have restricted its clinical prescription (Cao et al., 2021).

Due to the limitations of the aforementioned therapies, there have been continuing efforts to discover, develop, research, and implement new therapies for IS (Marquez-Romero et al., 2020; Williams et al., 2020). A number of natural ingredients and traditional herbal medicines have been investigated as potential therapies for IS, including ginsenoside Rg1 and Rb1 (Gao, Bai et al., 2020), *Naoxinqing* (NXQ) (Bei et al., 2004), *Buyang Huanwu* Decoction (Hao et al., 2012), saffron (SF) (Sharma et al., 2020) and related formulas including *Naodesheng* (Sugawara and Chan, 2003; Hao et al., 2015), *Weinaokang* (Zhang et al., 2010), also known as *Sailoutong* (SLT) (Fan et al., 2021). SLT is a standardized combination of SF that has been shown to be effective for vascular dementia in Phase I and Phase II clinical trials and is currently under the phase III trial for vascular dementia (Chang et al., 2016; Jia et al., 2018; Steiner et al., 2018). NXQ and SLT have shown vigorous antioxidant activity that may play a role in their neuroprotective effects (Bei et al., 2004; Bei et al., 2009; Fan et al., 2021). Ginsenoside Rg1 was demonstrated with a protective effect in animal models against ischemia/reperfusion (I/R) induced injuries (Xie et al., 2015b; Chen et al., 2019; Gao et al., 2020) possibly *via* alleviating blood-brain barrier (BBB) disruption (Zhou et al., 2014), downregulating inflammatory mediators (Zheng et al., 2019) and ameliorating protease-activated receptor-1 expression (Xie et al., 2015a). Additionally, Gj-4, a CR enrichment extract from *Gardenia Jasminoides J. Ellis* improved neurovascular protection, mitigating endothelial cell damage (Yang, 2020) and protecting memory deficit in rodents focal cerebral ischemia (Li et al., 2014; Pang et al., 2020; Liu et al., 2021a).

*C*. *sativus* L*.* (Iridaceae) in the superorder of monocots and subdivision of spermatophytes (Crocus-sativus, 2010) yields saffron (SF—the dried stigma) which demonstrated various pharmacological effects, including aphrodisiac (Kashani et al., 2013), anticonvulsant (El Khoudri et al., 2021), antitussive, and antianxiety (Khazdair et al., 2019). SF has long been used as a folk medicine to treat a variety of diseases and conditions, including neurodegenerative diseases, memory disorders, atherosclerosis, hyperlipidaemia, diabetes, high blood pressure, ulcers, and fatty liver disease (Abe and Saito, 2000; Sheikhani et al., 2017; Awasthi and Kulkarni, 2020). *C. sativus* L*.* is known to be native to Greece and Iran, and has been extensively cultivated in other countries such as southern Europe, Tibet, and India (Jan et al., 2014). The global production of SF is expected to increase by 12.09% in 2020–2027 (Kothari et al., 2021b), with 90% from Iran (Dhar and Mir, 1997; Kothari et al., 2021a). Iran has been reported as a country with a highly sustainable cultivation source of *C. sativus* L. based on climatic and edaphic conditions, and production and processing practices (Ghorbani and Koocheki, 2017). CR compounds can also be obtained from other sustainable species. For example, an extraction containing 17% of CRs can be obtained from *Gardenia Jasminoides* (Sommano et al., 2020). In addition petals of *C. sativus* L*.* which are considered a waste product in saffron production, may also be a sustainable source of CR (Zeka et al., 2020) with an estimated 0.6% (w/w) of CR can be recovered from dried petals (Zeka et al., 2015).

More than 100 compounds have been identified from SF, mainly terpenes, flavonoids, and anthraquinones (Chang et al., 2013), including CRs and crocetin (CC), which are responsible for the color of SF (Akbari et al., 2018; Kermanshahi et al., 2020; Csupor et al., 2021; Pandita, 2021). Given that CR is the main active ingredient of SF and SF-containing products such as SLT, it is of prime interest to review the current evidence for CRs in IS and related conditions. The effect of SF on IS has recently been briefly reviewed, although CRs and related compounds were not covered in detail (Azami et al., 2021). Thus, the focus of this review is to evaluate the current evidence on CR and associated analogues for IS, including pre-clinical and clinical studies, molecular mechanisms, toxicity, and safety, as well as current gaps and future directions.

## Literature Search Strategy

**FIGURE 1:**
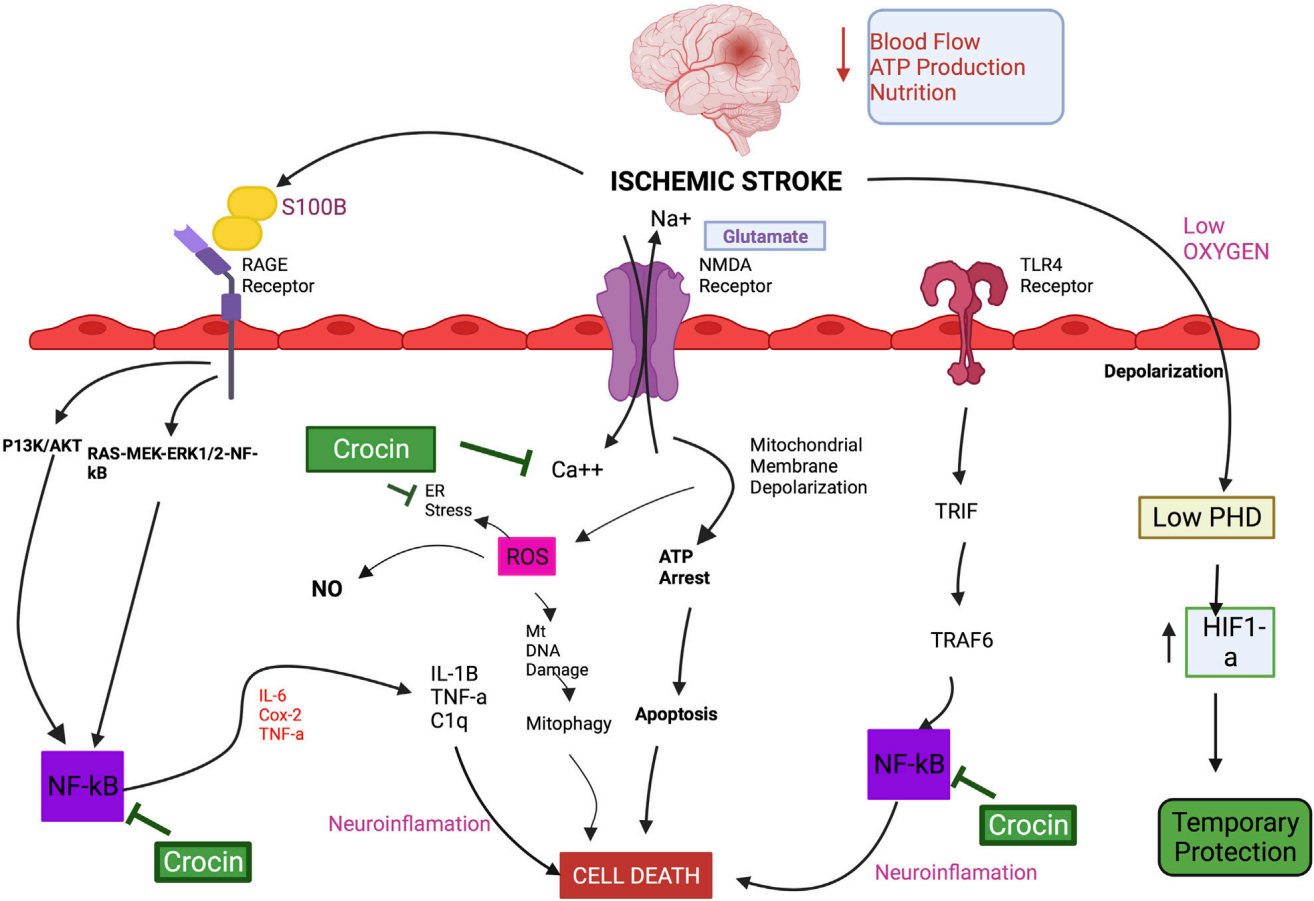
Schematic diagram of the mechanistic actions of crocin on the molecular pathways involved in IS.

Electronic databases including PubMed, Cochrane Library, Medline, Embase, Scopus, China National Knowledge Infrastructure (CNKI), and Web of Science were searched for relevant studies from their inception to 16 February 2022. The search terms include “crocin and analogues,” AND “Saffron” OR “*Crocus sativus* L*.*” AND “stroke” OR “ischemic stroke” AND “clinical trials” AND “pharmacokinetics,” “crocin” AND “neurons, astrocytes, microglial cells” OR “neuroprotection” OR “cytokines” OR “neuroinflammation” OR “neurotoxicity” OR “antioxidant” OR “apoptosis” OR “signaling pathways” OR “molecular targets” OR “mitochondria” OR “pharmacokinetics” OR “acute/chronic toxicity” OR “clinical trial,” OR “nanoparticle formulation,” “safranal,” “picrocrocin,” “crocetin” and their combinations. 287 research items including peer-reviewed papers, websites, and Chinese data-related research papers were considered suitable according to the review search criterion. Our inclusion criterion was all primary studies involving ischemic stroke, CR, and related compounds OR stroke OR ischemia OR hypoxia. All items identified were screened for relevant references excluding duplicates and non-peer-reviewed articles. Chemical structures of CR compounds were drawn using ChemDraw software (Table 1). Structural information of CRs such as isomeric SMILE was retrieved from PubChem and SciFinder Databases. Human metabolomics data were retrieved from the Human Metabolome Database v4 (HMDB) Canadian Database System (Wishart et al., 2018). All the diagrams (Figure 1) have been created with Adobe Illustrator (Adobe Inc., 2019), Preview MacOS v10.0 (944.5) and BioRender.com.

**TABLE 1:** CR and related compounds of SF.

Compound	CAS reg no.	Structure	Synonym	M. W (g/Mol)	MF	Isomeric SMILE	Ref.
CR	42553-65-1	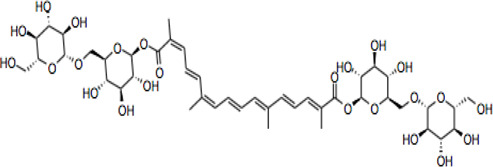	Crocin I; alpha-crocin; CR1, CR	977	C_44_H_64_O_24_	C/C(=C\C=C\C=C(\C)/C=C/C=C(/C)\C(=O)O[C@H]1[C@@H]([C@H]([C@@H]([C@H](O1)CO[C@H]2[C@@H]([C@H]([C@@H]([C@H](O2)CO)O)O)O)O)O)O)/C=C/C=C(\C)/C(=O)O[C@H]3[C@@H]([C@H]([C@@H]([C@H](O3)CO[C@H]4[C@@H]([C@H]([C@@H]([C@H](O4)CO)O)O)O)O)O)O	(Hadizadeh et al., 2010; Alavizadeh and Hosseinzadeh, 2014a)
CR 2	55750-84-0	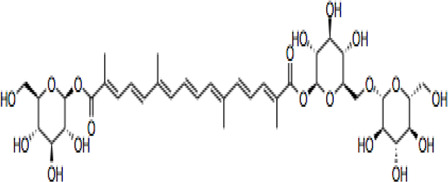	Crocin II; tricrocin, trans-crocin 3, crocin B, CR2	814.8	C_38_H_54_O_19_	C/C(=C\C=C\C=C(/C)\C=C\C=C(/C)\C(=O)O[C@H]1[C@@H]([C@H]([C@@H]([C@H](O1)CO[C@H]2[C@@H]([C@H]([C@@H]([C@H](O2)CO)O)O)O)O)O)O)/C=C/C=C(\C)/C(=O)O[C@H]3[C@@H]([C@H]([C@@H]([C@H](O3)CO)O)O)O	(Pfister et al., 1996; Wang et al., 2009)
CR 3	55750-85-1	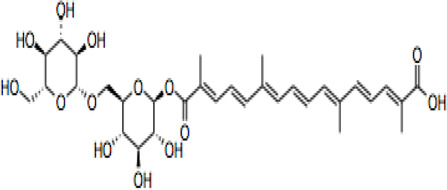	Beta-D-gentiobiosyl crocetin; crocin C, CR3	652.7	C_32_H_44_O_14_	C/C(=C\C=C\C=C(/C)\C=C\C=C(/C)\C(=O)O[C@H]1[C@@H]([C@H]([C@@H]([C@H](O1)CO[C@H]2[C@@H]([C@H]([C@@H]([C@H](O2)CO)O)O)O)O)O)O)/C=C/C=C(\C)/C(=O)O	(Chen et al., 2008; Lech et al., 2009)
CR 4	55750-86-2	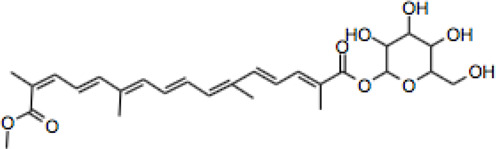	Crocin IV, CR4	504.6	C_27_H_36_O_9_	C/C(=C\C=C\C=C(\C)/C=C/C=C(\C)/C(=O)OC1C(C(C(C(O1)CO)O)O)O)/C=C/C=C(/C)\C(=O)OC	(Zhang et al., 2001; Karkoula et al., 2018)
CR 5	174916-30-4	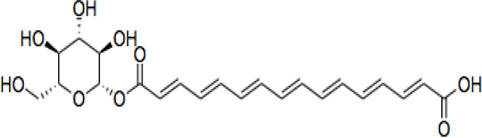	Trans crocin 5, CR5	434.4	C_22_H_26_O_9_	C([C@@H]1[C@H]([C@@H]([C@H]([C@@H](O1)OC(=O)/C=C/C=C/C=C/C=C/C=C/C=C/C=C/C (=O)O)O)O)O)O	(Zhang et al., 2001; Hadizadeh et al., 2010)
CR 6	164455-25-8	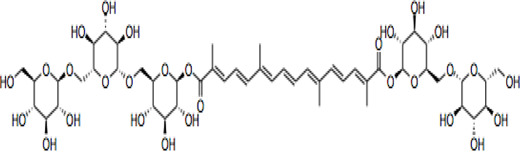	(13Z)-8,8'-Diapo-Psi, Psi-Carotene-8,8'-Dioic Acid 8-[6-O-(6-O-beta-D-glucopyranosyl-beta-D-glucopyranosyl)-beta-D-glucopyranosyl]8'-(6-O-beta-D-glucopyranosyl-beta-D-glucopyranosyl) ester, CR6	1139.1	C_50_H7_4_O_29_	C/C(=C\C=C\C=C(/C)\C=C\C=C(/C)\C(=O)O[C@H]1[C@@H]([C@H]([C@@H]([C@H](O1)CO[C@H]2[C@@H]([C@H]([C@@H]([C@H](O2)CO[C@H]3[C@@H]([C@H]([C@@H]([C@H](O3)CO)O)O)O)O)O)O)O)O)O)/C=C/C=C(\C)/C(=O)O[C@H]4[C@@H]([C@H]([C@@H]([C@H](O4)CO[C@H]5[C@@H]([C@H]([C@@H]([C@H](O5)CO)O)O)O)O)O)O	(Carmona et al., 2006; Hadizadeh et al., 2010; Verma and Middha, 2010)
CR 7	864547-06-8 (Unspecified)	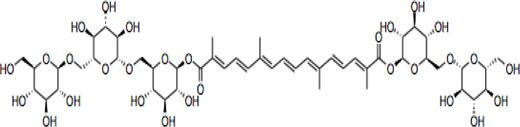	CR7	1301.2	C_56_H_84_O_34_	C/C(=C\C=C\C=C(\C=C\C=C(\C(=O)O[C@@H]1O[C@@H]([C@H]([C@@H]([C@H]1O)O)O)CO[C@@H]2O[C@@H]([C@H]([C@@H]([C@H]2O)O)O)CO[C@@H]3O[C@@H]([C@H]([C@@H]([C@H]3O)O)O)CO)/C)/C)/C=C/C=C(/C(=O)O[C@@H]4O[C@@H]([C@H]([C@@H]([C@H]4O)O)O)CO[C@@H]5O[C@@H]([C@H]([C@@H]([C@H]5O)O)O)CO[C@@H]6O[C@@H]([C@H]([C@@H]([C@H]6O)O)O)CO)\C	(Zougagh et al., 2005; NCBI, 2021)
	101124079 *(PubChem CID)						
CC	27876-94-4	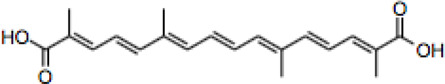	Transcrocetin; transcrocitinate, CC	328.4	C_20_H_24_O_4_	C/C(=C\C=C\C=C(\C=C\C=C(\C(=O)O)/C)/C)/C=C/C=C(/C(=O)O)\C	(Giaccio, 2004; Jackson et al., 2021; Qin et al., 2021)
Dimethyl Crocetin	5892-54-6	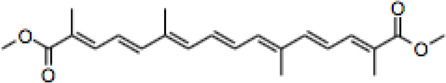	Crocetin dimethyl ester; gamma-crocetin, DMCC	356.5	C_22_H_28_O_4_	C/C(=C\C=C\C=C(\C=C\C=C(\C(=O)OC)/C)/C)/C=C/C=C(/C(=O)OC)\C	(Frederico et al., 2003; SUN et al., 2012)
Dicrocin	57710-64-2	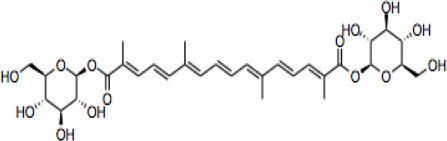	Crocetin diglucosyl ester; constitutional isomer of CR 3	652.7	C_32_H_44_O_14_	C/C(=C\C=C\C=C(\C=C\C=C(\C(=O)O[C@@H]1O[C@@H]([C@H]([C@@H]([C@H]1O)O)O)CO)/C)/C)/C=C/C=C(/C(=O)O[C@@H]2O[C@@H]([C@H]([C@@H]([C@H]2O)O)O)CO)\C	(Girme et al., 2021; Moratalla-López et al., 2021)

## Crocin and Related Compounds

Crocins (CRs) are a group of carotenoid compounds isolated from SF that also contain other active compounds, including picrocrocin and safranal (Suchareau et al., 2021). Seven different natural CR analogues have been identified from SF, namely CR (crocin 1, CR, CR1), CR 2 (crocin 2, CR II), CR 3 (crocin 3), CR 4 (crocin 4), CR 5 (crocin 5), CR 6 (crocin 6), and CR 7 (crocin 7) (Zhang et al., 2004; Mehrnia et al., 2017; Song et al., 2021b). All are mono or di glycosyl polyene esters of CC (Mohajeri et al., 2010a; Nam et al., 2010; Pang et al., 2020). CR and CR2 are the disaccharide analogues, whilst CR3 and CR4 are the monosaccharide analogues, which are more potent than CR and CR2 because of their structural orientations (Ulbricht et al., 2011). The biosynthesis of CRs is related to several enzymes such as cytochrome p450 (Gao et al., 2021), carotenoid cleavage dioxygenase (CCD), UDP-glycosyl transferase (UGT), and aldehyde dehydrogenase (Nagatoshi et al., 2012; Liu et al., 2020; Pu et al., 2020).

CR is one of the main bioactive ingredients in SF (Kothari et al., 2021b). The level of CRs in SF depends on their origin and quality. A high-quality SF contains about 30% of CRs (Azami et al., 2021). It was shown that Spanish SF contains more CRs, especially CR (9%), than that from other sources (Li et al., 1999). According to the Chinese Pharmacopeia, the total content of CR and CR2 in dried SF used in traditional Chinese medicine shall not be less than 10.0%, and the content of picrocrocin shall not be less than 5.0% (PPRC, 2020). On the other hand, dicrocin and CR3 are the isomers. Tricrocin, CR II or CR 2 are synonyms with the same molecular structure amidst isomeric structural orientation differentiations. To date, studies on CR3, CR4, CR5, CR6, and CR7 are still lacking. Table 1 shows the chemical structures of CR and related compounds.

CR molecule embodies two D-gentiobiose moieties (Mohajeri et al., 2010b; Ebrahim-Habibi et al., 2010). Studies on its structural activity relationship (SAR) revealed that the sugar moieties of CR related to its antioxidant activity and water solubility (Akhtari et al., 2013; Rahaiee et al., 2015; Akbari et al., 2018). Gentiobiose terminus is known to be involved in the conversion of CR to CC in enterocytes (Singla and Giliyaru, 2011). On the other hand, some synthetic CR analogues have been developed. For example, a-glucosyl-(1-6)-trans CRs have been shown with improved water solubility, and antioxidant and neuroprotective activities in mouse neuronal cell line (HT22) neuronal cells (Mok et al., 2020).

CC is a carotenoid (lacking provitamin functionality) recognized by diterpenic and symmetrical structure along with seven double bonds, four methyl functional groups, and two carboxylic groups (Giaccio, 2004) (Table 1). Its sodium salt, trans-sodium crocetinate (TSC), has been developed as a potential drug candidate (Gainer, 2000; Xi and Qian, 2006; Gainer, 2008; Wang et al., 2014; Shah et al., 2021). CC and TSC have similar pharmacological activities as CR (see below). Some bioactive synthetic analogues of CC have also been synthesized such as the diamide derivative of CC (Gao et al., 2017).

## Pharmacological Actions of Crocin and Related Compounds Against Ischemic Stroke

### Effect on Experimental Ischemic Stroke

The injuries caused by cerebral ischemic and IS are mainly caused by oxidative and nitrosative stress, and are also related to inflammation, apoptosis, BBB dysfunction, and edema formation, which increases the intracranial pressure and decreases the cerebral blood perfusion of ischemic areas (Akbari et al., 2018). The commonly used animal models of cerebral ischemia and IS injuries include middle cerebral artery occlusion (MCAO) in rodents to mimic the I/R injuries in humans after stroke. A number of studies have demonstrated the protective effects of CRs *in vitro* and *in vivo* (Table 2). Similar findings were reported by a group showing the protective effects of CR (20 mg/kg) and *Weinaokang* (10 and 20 mg/kg, which contains CR), against cerebral microvessel injury induced by global ischemia (Zheng et al., 2010). Vakili et al. (2014) reported CR (30, 60, and 120 mg/kg, i.p., given at the start of ischemia) dose-dependently decreased the infarct volume of cerebral I/R injuries. They also found that CR (60 mg/kg, given 1 h before, at the start, or 1 h after ischemia) reduced brain edema by 48%, 52%, and 51%, respectively. Sarshoori et al. (2014) showed in MCAO rats that CR (50 and 80 mg/kg, p.o.) reduced the cortical infarct volume by 48%–60%, and decreased striatal infarct volume by 45%–75%, respectively, with improved neurological deficit scores and decreased number of eosinophilic (prenecrotic) neurons,fiber demyelination and axonal damage in ischemic regions. Similar findings were obtained by Oruc et al. (2016) who used a global cerebral I/R model in rats (bilateral occlusion for 30 min followed by 30 min of reperfusion) and demonstrated that CR (40 mg/kg/day, orally) reduced the histopathological changes and apoptosis, and improved tissues oxidative index (Oruc et al., 2016). CR (10, 20, and 40 mg/kg/day i.p., given 7 days before the operation) attenuated the brain injury compared to that of the model group, with improved symptom score, attenuated brain edema, and improved pathological morphological and structural changes (Finley et al., 2017). Furthermore, CR was also shown to protect the BBB function during cerebral hypoxia/ischemia (Zhang et al., 2017). Similarly, Huang et al. (2019) demonstrated that CR at 50 and 100 mg/kg (p.o. for 7 days) decreased infarct volume and neurological scores in MCAO rats. Bin Wen et al. (2020) found significant results, stating that CR protected brain tissue against cerebral I/R damage, and that this effect was linked to its anti-oxidant and anti-inflammatory properties.

**TABLE 2:** Bioactivity of CRs *in vitro* and *in vivo.*

Compound	Animal/cell line	Type of study	Treatment	Effect	Mechanism	Reference
CR	Rat microglia	*In vitro*	10–50 µM	Antioxidant	Inhibited ROS production	(Mehri et al., 2011; Rao et al., 2019a)
	Mice brain slices	*In vitro*	10–20 µM	Anti-inflammation	Inhibited cytokines such as TNF-α, ROS, NO, and IL-1β	(Nam et al., 2010; Salama et al., 2020)
	Mice/rat brain	*In vitro*	0.5–2 µM/3 h	Anti-apoptosis	Inhibited Ca^2+^ overload inhibited ROS	(Mehri et al., 2011; Wang et al., 2019)
	Pheochromocytoma cell line-12 (PC-12 cells)/rats	*In vitro/in vivo*	10 mg/kg		Inhibited mRNA expression of p38, CASP-3 lowers brain damage, ROS	(Nam et al., 2010; Huang and Jia, 2019a)
	Pheochromocytoma cell line-12 (PC-12 cells)	In vitro	10 µM/6 days		Inhibited peroxide lipids	(Ochiai et al., 2004a; Wang et al., 2019)
					Regulated SOD level	
	Pheochromocytoma cell line-12 (PC-12 cells)	*In vitro*	10 µM		Inhibited TNF-α	(Soeda et al., 2001; Huang and Jia, 2019a)
					Inhibited caspase-3	
	Mouse neuroblastoma neuro-2a (N2a)/APP695swe cells.	*In vitro*	100–200 µM		Inhibited ROS	(Ochiai et al., 2004a; Du et al., 2021)
					Inhibited caspase-3	
					Inhibited cytochrome release, reduced apoptosis	
	AMI rats	*In vivo*	15 and 30 mg/kg	Decreased mitochondrial stress	Increased viability of mitochondrial respiratory enzymes, Increased ATP, Increased Na + -K + -ATP enzyme and Increased Ca^2+^-ATP enzyme and Inhibited mitochondrial Ca^2+^	Leijiao Fan, (2021a)
	Adult albino mice (CFT-Swiss mice)	*In vivo*	25 mg/kg/day; i.p.	Reduced rotenone-induced neurotoxicity; improved performance of mice in behavioral tests.	Inhibition of ROS generation; an increase of antioxidant enzymes activities; modulation of mitochondrial function; restored levels of dopamine, α-synuclein, and AChE activity in the striatum	Rao et al. (2019a)
	Wistar rats	*In vivo*	30 mg/kg/day; i.p.	Improved behavioral tests, Increased dopamine level in striatum	Activation of PI3K/Akt/mTOR pathways and enhanced miRNA-7 and miRNA-	Salama et al. (2020)
	Diabetic rats	*In vivo*	10^−9^ –10^−5^ mol/L	Downregulated vasoconstriction	Increased endothelial nitric oxide synthase	Sai Li, (2017)
	Hemorrhagic shock rats	*In vivo*	60 mg/kg	Antioxidant properties	Increased MDA in the lungs, kidneys, and liver	Yang (2020)
	Rat	*In vivo*	50 mg/kg	Neuroprotection	Suppresses caspase-3	Chen et al. (2015)
	Rat	*In vivo*	15, 30, 60, 120 mg/kg, I.p	Neuroprotection; Lowered infarct size	Inhibited MDA and increased GPx and SOD	Vakili et al. (2014)
	Rat hippocampus	*In vivo*	12.5, 25, and 50 mg/kg/21 day, I.p	Antidepressant effect	Increased BDNF and CREB levels	Vahdati Hassani et al. (2014)
	Rat	*In vivo*	40 mg/kg/day orally for 10 day	Neuroprotection	Suppressed ROS, HIF-1a and caspase-3	Oruc et al. (2016)
	Rat	*In vivo*	50, 100, and 200 mg/kg Ip	Neuroprotection	Improved biochemical indices, and enzyme level	Hariri et al. (2010)
	Rat	*In vivo*	10, 20, and 40 mg/kg, i.p. once/day	Neuroprotection	Suppressed ROS, increased MDA, SOD, and CAT activities, and inhibited cytokines including TNF, IL-1β, and IL-6 and IFN-γ	Bin Wen et al. (2020)
	Bovine Aortic endothelial cells (EC)	*In vivo*	25, 50, 100 mg/kg/day	Antiatherosclerosis	Decreased LDL and EC apoptosis; lowers MDA, NO, and Intracellular Ca^++^	He et al. (2005)
	Rat	*In vivo*	4.84, 9.69, and 19.38 mg/kg for 5 days	Antioxidant	Decreased total cholesterol, Triglycerides, SOD, CAT, GSH, MDA, and alkaline phosphatase	(Asdaq and Inamdar, 2010)
	Rats	*In vivo*	50 and 150 mg/kg	Antihyperglycemic	Lowers HbA1c, fasting blood glucose (FBS), and Upregulated blood insulin level	Kianbakht and Hajiaghaee, (2011)
CR & CR 2	Rat I/R Injury Model	*In vivo*	50 mg/kg	Neuroprotection	ROS suppression, decreased Bcl2, Bax, caspase 3, P38, NFkB, and increased total SOD	Lv et al. (2019)

TNF-a, tumor necrosis factor-alpha; OGD, oxygen glucose deprivation; iSOD, intracellular superoxide dismutase; NO, nitric oxide; ROS, reactive oxygen species; AMI, acute myocardial infarction; CREB, cAMP response element-binding protein; BDNF, brain-derived neurotrophic factor; NFkB, nuclear factor kappa light chain enhancer of B cells; IL-6, interleukin-6; ATP, adenosine triphosphate; AChE, acetylhydrolase; HIF-1a, hypoxia inducible factor-1a.

In addition, the curative effect of CR has also been demonstrated. Zheng et al. (2007) used a transient global cerebral ischemia mice model and demonstrated that CR (5, 10, and 20 mg/kg, given intragastrically from day 0 to day 21 after ischemia) significantly improved the capillary integrity and reduced mitochondria damage caused by I/R. Ochiai et al. (2004) reported that CR (10 mg/kg, IV, administrated immediately and 3 h after MCAO) significantly reduced the infarct volume in mice. They also showed that CR was more effective than other CR analogues and SF compounds (tricrocin, dicrocin, and picrocrocin) in promoting the expression of γ-glutamylcysteinyl synthase (γ-GCS) mRNA. γ-GCS is involved in the *de novo* synthesis of glutathione (GSH) as a rate-limiting enzyme reaction and plays a crucial role in IS (Su et al., 2020; Wu et al., 2020). CR (10 mg/kg) has also been shown to reduce the brain edema and infarct areas induced by hypoxia-ischemia when given immediately or after hypoxia-ischemia in post-natal C57BL/6J mice (Huang et al., 2019). Furthermore, it has been reported recently that CR (30 mg/kg and 60 mg/kg, p.o. for 7 days, administrated after cerebral ischemia) improved the memory loss in a rat model of cerebral ischemia, which was linked to increased hippocampal acetylcholine (ACh) level and reduced apoptosis (Yuan et al., 2020), a finding similar to an earlier observation using CR (25 mg/kg, i.p.) and hydroalcoholic extract of SF (250 mg/kg, i.p.) in a rat model of vascular dementia after permanent bilateral ligation of the common carotid arteries (Hosseinzadeh et al., 2012).

A recent study reported that the cerebral-protective effects of CR against cerebral I/R injury may involve gut microbiota (Zhang et al., 2019). Using a rat transient MCAO model, the investigators showed that the oral administration of CR was more effective than IV injection in reducing infarct volume and improving neurological behaviour changes. Since CC was detected in plasma after oral administration of CR but not after IV injection of CR, and the orally administered CC showed similar protection to that of CR, it indicated the importance of gut microbiota in facilitating the transformation of CR into CC. This was confirmed by the finding that CR could be deglycosylated to CC in the gut content of normal rats, but not in pseudo-germ-free rats. Metabolomics studies also indicated that gut microbiota facilitated the transformation of CR into CC (Zhang et al., 2019).

CC has demonstrated similar pharmacological actions to CR, including neuroprotection, anti-oxidation, and anti-inflammation (Tseng et al., 1995; Li et al., 2018; Hashemi and Hosseinzadeh, 2019; Cerdá-Bernad et al., 2020) as well as protective against cardiac ischemic mitochondrial injury (Gao et al., 2017). Recently Liu et al. (2021) showed that CC (5–50 mg/L) protected hypoxia-induced cell injuries and inhibited apoptosis in cultured human U87 glioma cells, and CC (5,10, and 50 mg/kg) reduced the infarct size and apoptotic cell numbers in brain tissue and improved pathological status in rats. These effects were associated with modulation of miR145-5p, toll-like receptor 4 (TLR4) and nuclear factor kappa light chain enhancer of B cells (NFκB) (p65) (Liu et al., 2021b). CC and related oxygen diffusion-enhancing compounds have been recently reviewed (Shah et al., 2021). These compounds have been shown with properties that improve the diffusion of oxygen in plasma, thus increasing oxygenation in ischemic brain tissue (Manabe et al., 2010; Wang et al., 2014; Bahr-Hosseini et al., 2021; Shah et al., 2021). TSC has shown potential as a therapeutic drug for early stroke intervention reducing the infarct and hemorrhagic volume in rodent models of ischemic and hemorrhagic stroke (Lapchak, 2010; Wang et al., 2014; Shah et al., 2021). In obese MCAO mice, TSC (0.14 mg/kg) showed a significant improvement in neurological deficit and neuroprotective effects evidenced by lowered brain edema, MMP-2, MMP-9, and inflammatory cytokine markers in brain tissues (Deng et al., 2015).

### Neuroprotection

One strategy for developing new therapies for IS to target neuroprotective signaling pathways (Rodriguez et al., 2021; Liu et al., 2012). CR has been extensively studied for its neuroprotective effects *in vitro* and *in vivo* (Table 2) (Yuan et al., 2020). Studies have shown that CR and related compounds protected CNS neurons in various conditions. For example, CR was demonstrated to protect against PC-12 cells injury in rats by increasing the synthesis of GSH (Ochiai et al., 2004b; Soeda et al., 2007), nitric oxide (NO), and decreasing MMP (Zheng et al., 2007). It induced the proliferation and migration of neural stem cells and inhibited the apoptosis of neural stem cells in cerebral I/R conditions, the effect involved Notch1 signaling and inhibition of inflammatory factors (An et al., 2020). CC also modulated the amyloidogenic pathway and tau misprocessing in neuronal cells (Shah et al., 2021). In the retinal ganglionic cells (RGC), CR prevented apoptosis induced by ischemic injury (Qi et al., 2013). In diabetic rats, CR was shown to act as a neuroprotective agent by lowering the malondialdehyde (MDA) and xanthine oxidase levels in the brain and cerebellum tissue (Altinzo et al., 2014). In addition, CR dose-dependently inhibited the ischemic cerebral neuronal apoptosis of proinflammatory cytokines in the ischemic tissue (Oruc et al., 2016). Huo et al. (2012) investigated the neuroprotective effect of CR in mice with traumatic brain injury. It was found that the intraperitoneal injection of CR (50–200 mg/kg) markedly reduced brain edema and motor functional deficits after the traumatic brain injury induced by physical damage from cortical impact injury. The traumatic brain injury restricted the supply of blood and oxygen in the area which led to the accumulation of reactive oxygen species and subsequent neuronal death.

Recently, CR was found to protect against hippocampal neuron damage in IS (Wu et al., 2020) and dopaminergic neuron damage in 1-methyl-4-phenyl-1,2,3,6-tetrahydropyridine (MPTP)-induced Parkinson’s disease mouse models (Haeri et al., 2019). CR (25 mg/kg) has also been shown to protect neurons against neurotoxicity induced by rotenone, methamphetamine, and acrylamide in mice and Wistar rats (Rao et al., 2019b; Salama et al., 2020; Mehri et al., 2015; Shafahi et al., 2018), as well as improving neuronal survival in hypoxic ischemia related brain damage (Huang and Jia, 2019b). Another study showed that CR (30 mg/kg) alleviated apoptosis, neurodegeneration, and enhanced protection in rotenone-induced Parkinson’s disease rats *via* mammalian target of rapamycin (mTOR) pathway activation (Salama et al., 2020). CR, *via* its metabolite crocetin monoglucuronide (CM), was also shown to inhibit ACh activity as predicted by docking studies (Zhu et al., 2019b). In addition, CR improved gut microbiota in stressed mice and decreased serum levels of interleukin (IL-6) and necrosis factor—α (TNF-α) (Wu et al., 2020).

Both CR and CR2 have been shown to enhance neuronal survival by downregulating caspase-3 (Casp3) and Nfkb1 mRNA expression after hypoxic ischemic CNS amelioration (Lv et al., 2019). In addition, CR has been shown to reduce the neurological deficit in a heme oxygenase-1 (HO-1) knockout mouse model of intracerebral hemorrhage (Duan et al., 2019) and reduce cytotoxicity induced by lethal agents. In glutamate-damaged HT22 cells, CR improved cell viability, suppressed reactive oxygen species (ROS) accumulation, calcium ion (Ca^+2^) load, and apoptosis (Wang et al., 2019). In diazinon induced subacute toxicities, CR at 50, 100, or 200 mg/kg doses (i.p.) significantly ameliorated the adverse effect of diazinon on enzyme levels, and biochemical indices and downregulated the levels of S100 calcium-binding protein B (S100B) (Hariri et al., 2010). Interestingly, S100B is the prime secretary cytokine from astrocyte during metabolic stress and is related to astrocyte activation (Gerlach et al., 2006). Thus, CR may affect the function of astrocytes in its anti-IS action, although its exact effect is still not clear, it has been suggested that neuroprotection in the central nervous system (CNS) may involve astrocyte support (Teo and Bourne, 2018). Targeting astrocytic survival has been shown to lead to lowered neurodegeneration (Freitas-Andrade and Naus, 2016), especially in IS conditiosn (Becerra-Calixto and Cardona-Gómez, 2017).

### Anti-Neuroinflammation

There is strong evidence for the involvement of CRs anti-neuroinflammatory effects in their neuroprotective actions (Deslauriers et al., 2011; Ahmed et al., 2020). Studies have shown that CR inhibited the production of certain inflammatory mediators such as TNF-α and interleukin-1B (IL-1B) in microglial cells (Nam et al., 2010; Bin Wen et al., 2020). CR at a dose of 40 mg/kg significantly lowered levels of IL-6 and TNF-α in chronic restraint stress mice (Xiao et al., 2020), as well as inhibited IL-1B in depression-induced mice (Xiao et al., 2019). The anti-neuroinflammatory effect of CR was also shown in the methamphetamine-induced neurotoxicity model in rats (Shafahi et al., 2018), and inhibited inflammation-related microglial activation in mice (Fernández-Albarral et al., 2019), and also mitigated neuroinflammation in rat striatum (Eteghadi et al., 2021). CR (50 mg/kg and 100 mg/kg) inhibited lipopolysaccharide (LPS) induced neuroinflammation in rats, whilst the effect was not dose-dependent (Azmand and Rajaei, 2021). Furthermore, CR downregulated IL-1β, NO, IL-6, and TNF-α generation in rats with hemorrhagic shock (HS) and increased the level of IL-10 (Yang and Dong, 2017). IL-10 is an anti-neuroinflammatory cytokine expressed by immune cells (Saraiva and O’garra, 2010). The upregulation of IL-10 by CR is also supported by other studies (Bakshi et al., 2018; Badavi et al., 2020; Yousefi et al., 2021), although there is a contradictory finding that CR decreased IL-10 level (Dianat and Radan, 2014). The reason for this discrepancy is not clear. It may be related to experimental or disease conditions. CR (10 mg/kg) attenuated TNF-a, inducible nitric oxide synthase (iNOS), NFκB expressions during doxorubicin-induced nephrotoxicity in rats (Hussain et al., 2021). In addition, CR has also been shown to regulate cyclooxygenase-1 (COX-1) and COX-2 enzymes in LPS induced RAW264.7 cells (Xu et al., 2009). In 5XFAD (5X Familial AD) mice, CR (10 mg/kg/day) improved BBB integrity and lowered amyloid ß (Aß) associated neuroinflammation while this effect was accompanied by suppressing mitogen-activated protein kinase (MAPK) and NFκB but activating nuclear factor-erythroid factor 2 related factor 2 (Nrf2) pathways (Batarseh et al., 2017; Hashemzaei et al., 2020). The role of anti-inflammatory effect of CRs on neuronal pain has been recently reviewed, showing suppression of NFκB in turn downregulates the levels of IL-6, IL-10, IL-1β, and TNF-α (Hashemzaei et al., 2020). NFκB relates closely to IS involving inflammatory biosensors (Harari and Liao, 2010). Similarly, anti-neuroinflammatory activities of CC and TSC have also been demonstrated, including inhibiting the formation of proinflammatory mediators, such as NO and cytokines, and regulating NFκB pathway (Deng et al., 2015; Hashemi and Hosseinzadeh, 2019; Liu et al., 2021b; Shah et al., 2021).

### Antioxidant Activity

Numerous studies have demonstrated that endogenous antioxidant levels were lowered after acute IS due to oxidative stress (Ullegaddi et al., 2006), and increased cellular ROS levels could ultimately lead to mitochondrial injury and cell death (Shahbaz, 2017a; Shahbaz, 2017b). Hence, antioxidants have long been investigated as a potential therapy for reducing IS injury (Shirley et al., 2014; Cichoń et al., 2017). The antioxidant activity of CR and related compounds have been well established, with increasing superoxide dismutase (SOD) and GSH synthesis and activities in brain tissue (Bandegi et al., 2014; Chen et al., 2015; Mehri et al., 2015; Zhang et al., 2017a; Krishnaswamy et al., 2020; Yousefi et al., 2021), decreasing oxidized lipids and oxidative stress in PC-12 cells (Ochiai et al., 2004b; Soeda et al., 2007), and reducing hypoxia-induced cell damage (Javandoost et al., 2017; Bukhari et al., 2018; Ghaffari and Roshanravan, 2019). In human myoblast cells, 0.3 μM CR inhibited hydrogen peroxide (H_2_O_2_) induced toxicity accompanied by decreased ROS and increased antioxidant enzyme activity (Nassar et al., 2020). In addition, CR was shown to upregulate MMP-2 and MMP-9 protein expression which contributes to neuroprotection and maintenance of BBB integrity (Yang and Rosenberg, 2015; Zhang et al., 2017a). In particular, Vakili et al. (2014) showed that the anti-IS effect of CR was associated with increased SOD and glutathione peroxidase (GPx) activity and reduced MDA content in the ischemic cortex. Oruc et al. (2016) reported that CR-induced protection of cerebral I/R is associated with improved tissues oxidative index. CR was also shown to curb ROS in depression-induced mice (Xiao et al., 2019). In addition, a study indicates that the antioxidant effect of CR may contribute to its protection against organ damage in HS (Yang and Dong, 2017). Similarly, the antioxidant activity of CC and TSC have been demonstrated, including inhibition of ROS formation, improving antioxidant enzyme activities, and suppression of related mitochondrial apoptosis, and Aβ mechanisms (Hashemi and Hosseinzadeh, 2019; Shah et al., 2021). It has been reported that the free radical quenching activity of CR is weaker than SF, indicating that SF may contain other antioxidant constituents or has a synergistic effect among its ingredients (Asdaq and Inamdar, 2010). It was found that trans-crocin-4 exhibited more potent antioxidant activity than CC in the human brain cells, indicating the sugar molecules in CR may be important for its antioxidant activity and Aß fibril formation inhibition (Papandreou et al., 2006). In addition, SLT, a CR-containing formula was shown to reduce H_2_O_2_ related injury in EA hy926 cells (Seto et al., 2017). However, it is not clear if this effect of SLT relates to CR.

### Cardiovascular Protection

Cardiovascular function is important for blood supply to the brain, thus the effect of CRs on cardiovascular functions may affect or contribute to their effects against IS. CR has demonstrated anti-ischemia effects in cardiac and vascular tissue. For example, CR (20 mg/kg) was shown to exhibit cardioprotective activity in I/R-related myocardial injury and decreased infarct size in ischemia the rats’ hearts by altering antioxidant status (Jahanbakhsh et al., 2012; Dianat et al., 2014). CR (100 mg/kg/day) attenuated cardiac inflammation and improved antioxidant capacity in female rats (Kocaman et al., 2021). Additionally, a study using cardiac ischemic rats showed CR at 20 mg/kg/day i.p. protected cardiac injury and increased SOD, MDA, and GSH antioxidant markers (Jahanbakhsh et al., 2012). Another study found that pre-treatment with CR followed by I/R (2 h hypoxia, 4 h reoxygenation) protected myocardial ischemic injury and regulated autophagy AMP-activated protein kinase (AMPK) mechanistic pathway (Zeng et al., 2016). Fan et al. (2021) showed CR protected myocardial mitochondria injury and acute myocardial infarction in rats (Leijiao Fan, 2021b). CR (15 and 30 mg/kg) significantly attenuated mitochondrial damage by increasing the membrane potential and reducing the mitochondrial permeability transition pore openness. The mechanism was associated with increased viability of mitochondrial respiratory enzymes, adenosine triphosphate (ATP), Na^+^-K^+^-ATP enzyme and Ca^2+^-ATP enzyme, and reduced mitochondrial Ca^2+^ concentration (Leijiao Fan, 2021b). Similar activities have been reported for CC and TCS, including cardioprotective effects against I/R injury, inhibiting myocardial infraction and cardiac hypertrophy, reducing blood pressure, and inhibiting platelet aggregation (Hashemi and Hosseinzadeh, 2019; Shah et al., 2021). For example, in a myocardial I/R rats model TSC (50 and 100 µg/kg) significantly alleviated I/R-induced cell injury *via* the SIRT3/FOXO3a/SOD2 signaling pathway (Chang et al., 2019). CR and related compounds have also been shown to possess vascular protective activity which may also contribute to their anti-IS actions. For example, CR improved vasodilation by acting on endogenous NO and endothelial NO synthase (Sai Li and ding, 2017) and reduced I/R injury in mice cerebral microvessels (Zheng et al., 2007). CC (25 and 50  mg/kg/day) was also shown to protect vascular function in stroke-prone spontaneously hypertensive rats (SHRSPs), with downregulated thrombogenesis, improved vasodilation, and endothelial function (elicits NO production), and antioxidant capacity (Higashino et al., 2014).

## Mechanism of Actions of Crocin and Related Compounds

### Potential Cellular Targets

CR has been shown to act on multiple brain cells including microglia, neurons, and astrocytes (Hosseinzadeh et al., 2014b; Zhu et al., 2019a). Table 2 shows the effects of CR and related compounds on different cells including microglia, endothelial cells and neurons (Table 2). For example, in syncytin-1-expressed primary human foetal astrocytes, CR (100, 200, and 400 µM) inhibited endoplasmic reticulum (ER) stress and nitric oxide synthase 2 and interferon gamma (IFN-α) expressions (Deslauriers et al., 2011). Although CR mitigated TNF-a release in astrocytes, microglia, and neurons after LPS stimulation (Gullo et al., 2017), the effect of CRs on astrocytes involving IS microvessels is still not clear. Astrocytes may play a critical role in neuroprotection during IS through their specialized functional and structural properties in the CNS (Liu and Chopp, 2016; Sun et al., 2019) including regulation of metabolic and homeostasis (Liddelow et al., 2017; Escartin et al., 2021). It is also necessary to investigate the effect of CRs on different phenotypes of astrocytes such as A1 (neuroinflammatory), A2 (neuroprotective), and A0 (nascent astrocytes).

### Potential Molecular Targets

As mentioned above, CRs have therapeutic action on multiple pathways, related to cell signalingsignaling, transportations, energy production, and redox homeostasis (Hosseinzadeh et al., 2014a), in particular neuroinflammation, antioxidation, and apoptosis mechanisms (Table 2). For example, CR inhibited hypoxia-inducible factor-1α which is an important molecular target in IS (Oruc et al., 2016) (Table 2). The action of CR on mitochondrial apoptosis is also important for their neuroprotective effects against IS (Li et al., 2021; Yousefsani et al., 2021), including casp 3, casp 8, casp 9, B-cell lymphoma-2 (Bcl-2), and Bcl-2 Associated X-protein (Bax) (Table 2). The key ROS-related enzymes targeted by CR and associated compounds or analogues include SOD, reduced nicotinamide adenine dinucleotide phosphate (NADPH) oxidase, and GPx (Bandegi et al., 2014; Chen et al., 2015; Zhang et al., 2017b). In addition, it has been suggested that matrix MMPs activation plays a role in IS (Yang and Rosenberg, 2015) and amelioration of MMP-2 and MMP-9 expressions by CR may provide neuroprotection and protection of BBB functions under cerebral ischemia (Zhang et al., 2017b). The key inflammatory signaling molecules influenced by CR and related compounds include TNF-α, IL-1β, IL-1α, IL-6, IL-1, and MMP-9, monocyte chemoattractant protein-1 (MCP-1), macrophage inflammatory proten-1α (MIP-1α), vascular adhesion molecule-1 (VCAM-1), E-selectin and fractalkine (CX3CL1) and related signaling pathways namely NFκB, MAPK, TLR4, c-Jun N-terminal kinase (JNK), and P38 (Che et al., 2001; Hussein et al., 2019; Barthels and Das, 2020). In addition, autophagy is another important cellular mechanism involved in various cellular functions (Shen et al., 2021), and is a significant adaptive mechanism in IS (Ajoolabady et al., 2021). It has been suggested that activation of autophagy is involved in the protective effect of CR against IS (Zeng et al., 2016) CR enhanced autophagy by downregulating the LC3-II/I and upregulating the p62 and mTOR expression in IS model of HT22 cells (Huang et al., 2019). CC and TSC have shown with similar activities affecting cellular redox signaling and inflammatory pathways, as well as regulating autophagy, which contribute to their anti-IS and neuroprotective pharmacological actions (Tseng et al., 1995; Hashemi and Hosseinzadeh, 2019; Cerdá-Bernad et al., 2020; Shah et al., 2021; Wani et al., 2021).

Table 3 shows the IS-related human proteins potentially regulated by CR, based on the human metabolomic data retrieved from the standard HMDB Canadian Database System. We identified 11 proteins as the possible targets of CRs, including S100β, vascular endothelial growth factor (VEGF), glial fibrillary acidic protein (GFAP) and compliment component 1q (C1q). Since S100B is elevated in patients after IS (Lasek-Bal et al., 2019) and higher S100B levels were observed in the hospitalized IS patients (Shotar et al., 2019), S100B may be a potential diagnostic and therapeutic biomarker of IS. Intracellularly S100B can act as a regulator of Ca^++^ homeostasis (Donato et al., 2009). Extracellularly, it can activate inflammatory and other signaling pathways, such as the mitogenic Ras-MEK-ERK1/2-NF-κB pathway. SF has also been shown to regulate glial GFAP, VEGF, and C1q in MCAO animals (Yang et al., 2019; Abdel-Rahman et al., 2020; Zhong et al., 2020), although the exact role of CR and related compounds in these actions is not clear. Further studies are mandatory to elucidate the mechanisms involved in the actions of CRs against IS and related conditions in human (Karkoula et al., 2020).

**TABLE 3:** IS-related human proteins targeted by CR, based on the metabolomic data by CRs (retrieved from the HMDB Canadian Database System).

Protein	Effect of CR	HMBD protein Id	Cellular location	Chromosome location	Main function	Metabolites
S100B	Downregulate	HMDBP07977	Nucleus and cytoplasm	Chr. 2	Involved in Ca^2+^ ion binding	Ca^2+^, Olopatadine
FIH-1	—	HMDBP01023	Nucleus (Potential),	Chr. 10	Oxygen sensing inhibits HIF1-a	Oxoglutaric acid, succinic acid, Fe^2+^, and CO_2_, O_2_, D-tartaric acid
VEGFA	Downregulated	HMDBP02130	Membrane	Chr. 6	Growth factor activity in angiogenesis and endothelial cell proliferation	Atorvastatin, pyroglutamic acid, heparin, and simvastatin
C1q	—	HMDBP02512	Secreted	Chr. 3	Involved in cytokine activity	Cyclic AMP
S100A10	—	HMDBP07984	—	Chr. 1	Ca^2+^ ion binding	Ca^2+^
Vimentin	Up-regulation	HMDBP01682	—	Chr. 1	Structural molecular activity	Carnosine
PHD1	Up-regulation	HMDBP09211	Nucleus and cytoplasm	Chr.19	Oxidoreductase activity	Ascorbic acid, L-proline, oxoglutaric acid, succinic acid, O_2_, and 4-hydroxyproline
NSE	Downregulate	HMDBP01086	Cell membrane	Chr. 12	Neuroprotective	Water, Ca^2+^, magnesium, 2-phospho-d-glyceric acid, phosphoenolpyrovic acid, and 3-dehydroquinic acid
MMP9	Downregulation	HMDBP02128	Secreted, extracellular matrix	Chr. 2	Metallopeptidase activity	Simvastatin, marimastat, Ca^2+,^ minocycline, Zinc, and Captopril
IL-1B (catabolin)	Downregulation	HMDBP02072	Secreted	Chr. 2	IL-1 receptor binding, inflammatory response	Minocycline
TNF-a (cachectin)	Downregulation	HMDBP02070	Enzymatic protein secreted	Chr. 6	TNFR binding	Butyric acid, isopropyl alcohol, glucosamine, atorvastatin, simvastatin, cis,trans-5′ hydroxythalidomide, chloroquine, clenbuterol, pranlukast, amrinone, and ethyl pyruvate
IL-6	Downregulate	HMDBP02087	Enzymatic protein secreted	Chr. 7	Cytokine activity, IL-6 receptor binding	Simvastatin

Chr, chromosome number; “-”, No data as per human metabolomic database; TNFR, tumor necrosis factor receptor.

## Pharmacokinetics of Crocins

The pharmacokinetics of CR and related compounds have been reviewed (Xi and Qian, 2006; Khorasany and Hosseinzadeh, 2016; Hosseini et al., 2018; Hashemi and Hosseinzadeh, 2019; Veisi et al., 2020; Song et al., 2021a; Shah et al., 2021).

Asai et al. (2005) studied the absorption of CR and CC in mice and showed that neither CR nor CR2 were detectable in the plasma after the oral administration of a mixed micelle solution containing CC or CRs, whereas CC was rapidly absorbed into the blood and detected in plasma as free form and glucuronide conjugates, indicating the metabolism of CRs mainly involves glucuronidation in the intestine and liver. Another study confirmed that CM (CR metabolite) was detected in blood and brain after oral administration of CR (Zhang et al., 2012). Xi et al. (2007) studied the absorption of CR and CC in rats after single or repeated oral doses (40 mg/kg by oral gavage), and found that CR was not detectable, while CC was present in the plasma at low concentrations. They also demonstrated that CR was excreted through the intestinal tract following oral administration, indicating the intestinal tract may serve as a site for CR *via* hydrolysis. Another study in stroke-prone spontaneously hypertensive rats found a high level of CC in plasma and brain after oral administration of 100 mg/kg CC (Yoshino et al., 2011). The elimination half-life (t1/2k) after an oral dose of CR (1 mg/kg) was reported as 3.0 ± 0.6 h (Zhang et al., 2012). Studies in humans confirm that CC is rapidly absorbed after oral administration (Hosseini et al., 2018). In healthy adult volunteers, it was reported that the peak plasm (Cmax) level of CC after receiving 7.5, 15, and 22.5 mg doses was 100.9–279.7 ng/ml, and the mean time to reach maximum concentration (T_max_) was 4–4.8 h, AUC0–24 h ranged from 556.5 to 1720.8 ng h/ml and the mean elimination half-life (T_1/2_) was 6.1–7.5 h (Umigai et al., 2011). The pharmacokinetics of CC after oral and IV administrations is consistent with a two-compartment model (Xi and Qian, 2006).

There is evidence that CR could not penetrate Caco-2 monolayers, while trans-crocetin permeated the intestinal barrier (Lautenschläger et al., 2015). It has been suggested that CRs are hydrolyzed in the intestine by intestinal cells to the deglycosylated trans-crocetin, which is subsequently absorbed by passive transcellular diffusion (Lautenschläger et al., 2015; Karkoula et al., 2018; Shah et al., 2021), most likely *via* gut microbiota mediated biotransformation as mentioned above (Zhang et al., 2019). This mechanism may be important for CRs to exert their anti-IS and neuroprotective actions in the brain, as CC has been shown to be able to permeate BBB and accumulate in the brain (Lautenschläger et al., 2015; Karkoula et al., 2018). On the other hand, some CRs such as trans-crocin 4 were shown to be able to across BBB in mice after ip administration despite its highly hydrophilic character (Lautenschläger et al., 2015; Karkoula et al., 2018). Studies showed that CC can be rapidly distributed into different tissues, including the liver and kidneys, partly due to its weak binding to plasma albumin (Xi and Qian, 2006; Hosseini et al., 2018; Christodoulou et al., 2019; Hashemi and Hosseinzadeh, 2019; Shah et al., 2021). A study on rats by Zhang et al., showed that T_1/2_ was estimated as 2.5–2.9 h after oral administration of different doses of CC (Zhang et al., 2017a). CR is excreted primarily through the intestinal tract in feces after oral administration (40 mg/kg), with 59.507% ± 13.56% excreted (Asai et al., 2005), and *via* urine after IV administration with a cumulative excretion fraction of 67.17% ± 4.79% within 48 h (Zhang et al., 2019).

### Nanoparticle Formulation and Green Synthesis

Nanoscale drug delivery is an important tool to improve the pharmacokinetics and bioavailability of drugs and natural drugs (Puglia et al., 2010). Nanoparticle (NP)-based drug delivery systems have shown advantages in improvised bioavailability, bioadhesion, and controlled drug release in the gastrointestinal (GI) tract (Kim et al., 2006; Dudhani and Kosaraju, 2010). For example, some chitosan-alginate biofilm forming agents have been used for pH-sensitive nanomolecular formulations to control drug movement across the GI tract (George and Abraham, 2006). Thus, NP-based formulations can be used to enhance the bioavailability and activity of CRs and CC (Shah et al., 2021). A nano-encapsulated formulation of chitosan alginate biofilm-forming agents has been shown with improved CR stability and bioavailability, and controlled release (Rahaiee et al., 2015) (Mirhadi et al., 2020). CR chitosan-alginate NPs also showed improved antioxidant and anticancer activities *in vivo*, suggesting potential therapeutic applications for these preparations (Rahaiee et al., 2017). Another recent study explored an NP-CR formulation with dextran/chitosan sulphate (DS/CH) coated NPs loaded with CR, and demonstrated its activity in downregulating VEGF and AB142 levels accompanied by a stronger antioxidant capacity in SHSY5Y cells (Song et al., 2022). In addition, a water-soluble crocetin-γ-cyclodextrin formulation significantly increased the bioavailability of CC and facilitated it crossing the BBB to enter the brain (Wong et al., 2020). Furthermore, CR-NPs have been formulated with polymeric carriers to improve the stability of CR (Mirhadi et al., 2020).

Green synthesis is an advanced method that uses natural reducing agents, plugs, and stabilizers surpassing the employment of toxic and expensive chemicals and high energy costs (Hussain et al., 2016). There is an increasing need for optimal eco-friendly and non-toxic methods of developing NPs such as gold NPs (AuNPs) preparation with CR (Hoshyar et al., 2016) and SF (Abootorabi et al., 2016). Solid lipid nanoparticles belong to the lipid nanotransporter family that can solubilize hydrophilic and lipophilic molecules in physiological environments. This is controlling their release and protects them from degradation (Tapeinos et al., 2017). CC and CR solid lipid nanoparticles can be prepared using Softisan 100 (hydrogenated coco glyceride) and Pluronic F68 (Poloxamer 188) with these lipid substrates commonly regarded as the best with low melting point (35°C), which is an important property related to the stability of NPs (Puglia et al., 2019). These nanoparticles have been tested in cancer cell lines and showed a prolonged antioxidant activity, and better antitumor cytotoxicity than free CR (Puglia et al., 2019).

### Clinical Evidence

A number of clinical studies on SF and CR have been conducted, involving healthy subjects or patients with various conditions such as metabolic syndrome (Kermani et al., 2017), depression, and coronary artery disease (CAD) (Abedimanesh et al., 2017). Table 4 summarizes the clinical studies on SF and CR. For example, a study with SF extract capsules (200 mg/day) showed that it was effective against IS with a long term (up to 3 months) neuroprotective effect, based on the National Institute of Health Stroke Scale (NIHSS), with improved Barthel index and brain-derived neurotrophic factor (BDNF) levels, and decreased stroke severity with lowered levels of serum neuron-specific enolase (NSE) and S100 (Asadollahi et al., 2019). A similar finding was obtained in an RCT involving 40 patients with acute IS, and revealed that SF at 400 mg/day decreased the severity of stroke as assessed by the NIHSS score, with an improved MDA level (Gudarzi et al., 2020). Other studies demonstrated the effectiveness of SF, in combination with Ritalin, for patients with attention deficit hyperactivity disorder (ADHD) (Pazoki et al., 2022), or CAD with a significant inhibition of circulating MCP-1 (Abedimanesh et al., 2020). In addition, a study showed that 30 mg of SF supplement for 16 weeks improved cognition function (change in both AD Scale-cognitive subscale (ADAS-cog) and clinical dementia ratings-scale sums of boxes (CDR-SB) in patients with mild to moderate Alzheimer’s disease (Akhondzadeh et al., 2010). This is supported by a recent systematic review of five RCTs involving 325 subjects on AD and mild cognitive impairment, suggesting that SF may be as efficacious as common drugs against AD, although it should be taken with caution as there may be an unknown or high risk of bias due to the low quality of some of the studies included (Avgerinos et al., 2020). On the other hand, a recent trial involving 50 patients with type 2 diabetes (T2D) confirmed a significant improvement in glycaemic control and insulin resistance after administration of 15 mg CR twice daily for up to 12 weeks (Behrouz et al., 2020). Furthermore, a double-blind RCT involving 62 participants with mild erectile dysfunction (ED) showed that administering 15 mg SF twice a day improved erectile function without obvious side effects, indicating it may be effective against ED, especially in patients reluctant to accept the prescription of phosphodiesterase type 5 inhibitors (Najafabadi et al., 2022). A small trial (40 patients) on depression compared the effect of SF (30 mg/day) and fluoxetine (40 mg/day) and found no significant difference between the two groups in reduction of Hamilton depression rating scale, and the frequency of adverse events, indicating SF has similar antidepressant activity as fluoxetine, although further research with larger sample size is needed (Shahmansouri et al., 2014). Currently, there is no published RCT on CR or related compounds for treating IS. Zhu et al. (2010) described earlier a study using a CR injection (40 and 80 mg/day for 2 weeks) treating 60 patients with thrombotic cerebral infarction, and showed a significant improvement in symptoms, with an overall effective rate of 91% and 80%, respectively, and without obvious side effects. Given that CR has been demonstrated with efficacy in various conditions (Table 4), further clinical trials on the effect of CR on IS are mandatory. On the other hand, TSC has been studied as a synthetic carotenoid drug to enhance oxygenation of hypoxic tissue in addition to the standard of care, including Covid-19 and a Phase 2 trial on efficacy and safety for suspected stroke. However, the stroke trial was terminated due to the COVID-19 pandemic, according to the information posted on Clinicaltrials.gov (Zhu, 2010; Shah et al., 2021; Streinu-Cercel et al., 2021).

**TABLE 4:** Clinical studies on CR and SF.

Testing agent	Study design	Treatment	Key finding	Reference
SF	RCT on IS (*n* = 39)	SF extract (200 mg/kg) for 4 days and 3 months follow-up	Lowered stroke severity, higher Barthel index, short- and long-term protective effect	Asadollahi et al. (2019)
		Placebo *n* = 20		
		SF treatment *n* = 19		
SF	RCT on IS	SF capsule 400 mg/day for 4 days	Lowered stroke severity on NIHSS score	Gudarzi et al. (2020)
		Placebo (*n* = 20)	Oxidative stress markers decreased	
		Treatment (*n* = 40)	Decreased NSE	
SF	Double-blind, RCT in healthy subjects (*n* = 60)	200–400 mg/day for a week	Not affecting coagulant or anti-coagulant system	Ayatollahi et al. (2014)
		Placebo (*n* = 20)		
		SF (*n* = 20)		
SF	Healthy subjects (*n* = 10)	200 mg/day		Modaghegh et al. (2008)
Safranal & CR (Affron)	RCT Placebo controlled trial on depression *n* = 128)	Placebo (*n* = 3)	Reduced anxiety and the doses were safe	Kell et al. (2017)
		22 mg/day (*n* = 41)	No side effects observed	
		28 mg/day (*n* = 42)		
CR	Placebo controlled trial in patient with metabolic syndrome *n* = 30)	15 mg/twice per day for 8 weeks placebo *n* = 30	Decreased Serum PAB	Nikbakht-Jam et al. (2016)
		treatment *n* = 30	No side effect	
CR	Placebo-RCT on diabetic maculopathy (*n* = 60); (*n* = 101 eyes)	5–15 mg/day tablet for 3 months	CR 5mg/day improves central macular thickness (CMT), HbA1c, and FBS levels	Sepahi et al. (2018)
		third group as Placebo (*n* = 34)	Some side effects reported such as feet swelling and polyphagia	
		CR 5 mg/day *n* = 34		
		CR 15 mg/day *n* = 33		
CR	Placebo RCT of MMT (methadone maintenance treatment) patient	CR, 15 mg twice a day, CR *n* = 25	Improved mental health status	Khalatbari-mohseni et al. (2019)
		Placebo, 15 mg twice a day, *n* = 25		
CR	RCT Methadone maintenance treatment (MTT) patients	15 mg/day for 8 days	Improved mental health and metabolic profile	Ghaderi et al. (2019)
		CR (*n* = 26), placebo (*n* = 27)		
SF	Alzheimer’s disease	30 mg/day capsule for 16 days	Improved cognitive function	Akhondzadeh et al. (2010)
CR	Healthy volunteers	42 healthy volunteers CR tablet 20 mg/day for a month (*n* = 22)		Mohamadpour et al. (2013)
		To check the safety profile	Lowered amylase and WBCs after 1-month treatment. No significant changes in kidney and liver functions. No major adverse events were observed	
CR	RCT	100 mg/day tablet for 6 weeks *n* = 24 placebo *n* = 24 CR	Lowered cholesterol and TG	Kermani et al. (2017)
	Metabolic syndrome			
CR	RCT	30 mg/8 weeks 45–55 years CAD patients	Reduced depression	Abedimanesh et al. (2017)
	CAD depression			
CR	RCT Metabolic Syndrome	30 mg/day for 8 weeks 44 patients *n* = 22 CR *n* = 22 placebo	Increased serum cholesteryl ester transfer protein, no effect on HDL, LDL, TG, FBG	Javandoost et al. (2017)

PAB, pro-oxidant–antioxidant balance; TG, triglyceride; LDL, low-density lipoprotein; HDL, high-density lipoprotein; FBG, fasting blood glucose; RCT, randomized controlled trial; CAD, coronary artery disease.

### Toxicity and Safety of Saffron, Crocin, and Related Compounds

The toxicity of SF has been well studied (Alavizadeh and Hosseinzadeh, 2014b; Abu-Izneid et al., 2020) with a lethal dose (LD_50_) ranging from 1 to 5 g/kg, indicating it is mildly toxic compared to nontoxic compounds (LD_50_ > 5 g/kg) (Abu-Izneid et al., 2020). A recent study on the acute toxicity of orally administrated SF showed its LD_50_ as 4.12 ± 0.55 g/kg in mice (Gupta and Pandey, 2020). SF at the therapeutic doses (30–60 mg/day) has been shown with certain side effects including hypomania, sedation, nausea, mild headache and anxiety, dry mouth, dizziness, vomiting, and fatigue after 6 or 22 weeks (Akhondzadeh et al., 2005; Akhondzadeh et al., 2010). In general, SF at 1.5 g/day has been considered to be safe with no significant adverse drug effects (Ghaffari and Roshanravan, 2019). At a high dosage of 10 g/day in humans, SF manifested abortion or life-threatening complications (Mollazadeh et al., 2015), such as temporary paralysis after hallucination and abortion accompanied by maternal morbidity (Moghaddasi, 2010; Ghorbani and Koocheki, 2017). In comparison, the LD_50_ for CR could not be obtained as it did not cause mortality in mice after i.p. administration at 0.5–3 g/kg (Hosseinzadeh et al., 2010). An early study found that a high dose of CR (100 mg/kg for 2 weeks) caused liver injury and black pigmentation in rats (Wang et al., 1984). However, a recent comprehensive study on the acute and subacute toxicity of CR (up to 3 g/kg po and i.p.) in rodent found it did not cause damage to major organs (Hosseinzadeh et al., 2010). Another study found that CR at doses of 50,100, and 200 mg/kg (once a week for 4 weeks, i.p.) in rats did not cause significant changes in liver enzymatic profile and non-pathological tissue changes (Hariri et al., 2010). Rats fed with 1% CR for 4 months showed a reversible pigmentation (Wang et al., 1984; Imran et al., 2019). These findings indicate that CR is generally safe and well-tolerated. However, CR at 200 and 600 mg/kg i.p. was found to affect skeleton formation in pregnant mice indicating it may have developmental toxicity at very high doses (Moallem et al., 2016). On the other hand, CC was reported with a teratogenic effect at high concentration (200 µM) in frog (Xenopus) embryos (Martin et al., 2002), although no genotoxicity was observed for CC in V79 Chinese hamster cells (Ozaki et al., 2002), and no retinal toxicity was observed for CC in rabbit eyes (Wang et al., 2019). CR and CC have been well demonstrated for their cytotoxic activity against several cancer cells (Hoshyar and Mollaei, 2017; Hashemi and Hosseinzadeh, 2019; Veisi et al., 2020).

The clinical safety of CR has been demonstrated in several human trials (Ahmed et al., 2020). In a randomized double-blind placebo-controlled trial in healthy volunteers (*n* = 42), CR at 20 mg/day administrated for 1 month showed a safe profile of CR, with only minor adverse reactions in conjunction with decreased partial thromboplastin time, amylase, and mixed monocytes, basophils, and eosinophils (Mohamadpour et al., 2013). Another double-blind placebo-controlled study in schizophrenic patients (*n* = 22) found that CR tablet (15 mg/twice a day) caused no side effects or significant changes in liver, kidney, and thyroid markers and hepatological components (Mousavi et al., 2015). A randomized trial on diabetic maculopathy diagnosed 60 patients, and showed that CR tablets (5 or 15 mg/day for 3 months) caused some minor adverse reactions including polyphagia (4 patients), foot swelling (2 patients ), burning of eyes (3 patient), red-eye (2 patients), subconjunctival hemorrhage (5 patients), eye swelling (3 patients ) and stomach ache (1 patient) (Sepahi et al., 2018). Another clinical trial on CR as an adjunct therapy to methadone against opioid withdrawal in 50 patients found that CR at 15 mg twice a day for 8 weeks caused some minor side effects including headache, insomnia, nausea, and dyspnoea (Khalatbari-mohseni et al., 2019). Similarly, CC at 7.5 mg/day for 14 days in a randomized, double-blind, placebo-controlled, crossover trial on sleep quality was found to improve subjective sleep quality and no obvious adverse events linked to CC intake were observed, indicating that CC at this dose and treatment protocol is safe (Umigai et al., 2011). A 12-week RCT involving 32 healthy adult volunteers showed that CC (7.5 mg/day) had no significant adverse effects (Yamashita et al., 2018) while increased delta power and enhanced the refreshing feeling while waking up (Umigai et al., 2018). Overall, CR and CC at common therapeutic doses are generally safe in humans. Animal toxicology studies and phase I clinical trials had been conducted for TSC and showed it was well tolerated and safe in humans, although no published data are available (Gainer, 2008). Nevertheless, Mohler III et al. (2011) showed that TSC at a dose range (0.25 to 2.0 mg/kg, IV, once daily for 5 days) was safe and well-tolerated in patients with peripheral artery disease (PAD).

## Further remarks and Conclusions

Significant progress has been made recently in understanding the actions of CR and related compounds on IS-related conditions and the mechanisms involved. The current pre-clinical and clinical evidence indicates a great potential of CR, CC, and TSC to treat IS and related conditions. However, these studies have some limitations and there are still gaps that curb the clinical translation of research findings. For example, although there is strong pre-clinical evidence for the beneficial actions of CR against IS, the current clinical trials on CR and related compounds are limited to other conditions, and specific clinical trials on IS are still lacking. In addition, many published studies are with small sample sizes and some are subject to potential bias, thus, high-quality clinical studies are needed to further confirm their efficacy and safety. It is also important to establish the proper treatment protocol and compare the effects of these compounds under short-term and long-term treatments. More research is needed to improve the bioavailability of CR, including developing formulations or delivery systems to maximize their efficacy and safety, with proper dosage regimen and route of administration. Further research is also needed to elucidate the mechanism(s) involved in the actions of CR and related compounds, including transformation and site of actions, molecular targets, and sensitive markers. Among these, the synergy of these compounds with other drugs or ingredients needs to be explored. For example, it has been shown that TSC, given in combination with rtPA either before or after embolization, improved the treatment outcomes in experimental acute IS (Lapchak, 2010). CR has also been shown with a synergistic effect with zinc sulfate to reduce hepatic I/R injury in Rats (Mard et al., 2017). Further research in this area will warrant the potential therapeutic value of these compounds not only as drug candidates by themselves but also as a complementary therapy with existing therapies and other drugs available. Finally, current studies have focused on CR and CC, more research on other forms of CRs is needed.

In conclusion CRs, most importantly CR, are key active compounds of *C. sativus* L. (SF). These compounds have been demonstrated with beneficial pharmacological actions in preventing or reducing IS-induced injury *via* multiple mechanisms including neuroprotective, antioxidant, anti-inflammation, and other cerebral protective activities. CR can act on various cellular and molecular mechanisms related to IS in particular neuroinflammatory mitochondrial signaling pathways, HIF1a, VEGF, and cytokines. CR has low bioavailability and its conversion to CC by gut microbiota may be important in mediating its therapeutic effect. Toxicological and clinical studies indicate that CR is generally safe in humans. The current evidence indicates that CR and related products may have potential as stand-alone or adjuvant therapy for treating IS, although further confirming clinical studies are needed. The elucidation of CRs molecular targets and synergistic mechanisms with other drugs or ingredients may help translate preclinical findings into novel therapies for the intervention, management, and prognosis of IS and related conditions.
